# Multiple linear regression model for improving accuracy of capsulorhexis size calculation in femtosecond laser-assisted cataract surgery for adults: a retrospective single-center study

**DOI:** 10.1186/s12886-023-02776-w

**Published:** 2023-01-11

**Authors:** Miki Akaishi, Takeshi Teshigawara, Seiichiro Hata, Akira Meguro, Nobuhisa Mizuki

**Affiliations:** 1 Department of Ophthalmology, Yokosuka Chuoh Eye Clinic, 2-6 Odaki-Cho, Yokosuka, Kanagawa 238-0008 Japan; 2 Tsurumi Chuoh Eye Clinic, Tsurumi, Yokohama, Kanagawa Japan; 3 Department of Ophthalmology, Yokohama City University School of Medicine, Kanazawa, Japan; 4 Yokohama Sky Eye Clinic, Yokohama, Kanagawa Japan

**Keywords:** Femtosecond, Laser, Cataract, Adult, Capsulorhexis, Linear regression, Intraocular lens, Retrospective, Optical coherence tomography

## Abstract

**Background:**

Differences between programmed capsulorhexis diameter and actual resulting capsulorhexis diameter (ARCD) are commonly encountered in femtosecond laser-assisted cataract surgery (FLACS). The purpose of this study was to identify the preoperative ophthalmic variables influencing capsulorhexis diameter index (CDI) in FLACS for adults and create a multiple linear regression model for obtaining a more accurate capsulorhexis diameter.

**Methods:**

This retrospective study involved sixty-seven eyes of 44 patients who received FLACS and intraocular lens implantation. The ARCD was measured using anterior segment swept-source optical coherence tomography (CASIA 2). Keratometry (K1, K2 and average K), anterior chamber depth (ACD), lens thickness (LT), anterior chamber width (ACW), white-to-white (WTW), curvature radius of anterior lens capsule (Front R) and axial length (AL) were all measured preoperatively. Based on the derived data, LT/ACW, LT/AL, LT/ACD and LT/ACW/Front R were calculated. The ratio of the programmed capsulorhexis diameter and ARCD was defined as the CDI. Correlation analysis was conducted to examine the relationship between preoperative variables listed above and the CDI. Multiple linear regression analysis was applied to select the most influential preoperative variables on CDI.

**Results:**

ACD, LT, ACW, Front R, AL, LT/ACW, LT/AL, LT/ACD, and LT/ACW/Front R showed significant correlation with CDI. Front R and LT/ACW/Front R were selected as constants in the multiple linear regression model using stepwise variable selection. The following equation represents the multiple linear regression model: CDI = 1.306–4.516 × LT/ACW/FrontR-0.011 × Front R, when *P* < 0.0001, adjusted R-squared = 0.919, variance inflation factor = 8.389, and Durbin-Watson ratio = 1.846.

Predicted postoperative capsulorhexis diameter (PPCD) equation was created based on CDI equation as follows: PPCD = programmed capsulorhexis diameter × 1.306–4.516 × LT/ACW/FrontR-0.011 × Front R.

**Conclusion:**

Front R and LT/ACW/Front R were found to be the most significant influencing factors of capsulorhexis size. CDI and PPCD calculation equations presented in this study may be useful in setting up more accurate programmed capsulorhexis diameter for FLACS in adults, resulting in a precise ARCD.

## Background

Continuous curvilinear capsulorhexis (CCC) is one of the most important processes in cataract surgery since the size of CCC can affect the position of intra ocular lenses (IOLs), postoperative refraction and incidence of postoperative capsule opacification [1–5]. However, it is challenging to manually create the same size of capsulorhexis consistently [6, 7]. Therefore, femtosecond laser assisted cataract surgery (FLACS) was designed to achieve a consistent circularity and size of capsulorhexis [8, 9]. Nevertheless, clinicians have often noticed that the actual resulting capsulorhexis diameter (ARCD) is different from the attempted (programmed) capsulorhexis diameter by comparing the size between optics of IOLs and capsulorhexis. In fact, previous clinical studies have demonstrated the deviation of ARCD from the programmed diameter in FLACS [10–13]. Thus, it may be useful to identify the preoperative variables that influence the ARCD. The aim of this study was to analyze these variables and to create a multiple linear regression model that can allow the derivation of a more accurate and constant capsulorhexis diameter during cataract surgery.

## Methods

This retrospective study enrolled patients who underwent cataract surgery via FLACS between September 2021 and April 2022 at the Yokosuka Chuoh Eye Clinic, Yokosuka, Kanagawa, Japan. The ethical committee of the Yokosuka Chuo Eye Clinic approved this study (reference number: 2022–002). The study adhered to the tenets of the Declaration of Helsinki throughout the data collection process. After a detailed explanation of the process and possible results, informed consent was obtained from all subjects.

Patients satisfying the following criteria were included: cataract in one or both eyes; no complication encountered during surgery, including anterior capsule tear and post-capsule rupture; no complications encountered during femtosecond laser treatment, including docking failure, eye movement, incomplete capsulorhexis, and capsulorhexis tags and tears; no medical history of ocular trauma and ophthalmic surgery; no corneal scarring and dystrophy; and no preoperative glaucoma. Patients with pupil diameter of less than 6 mm after the usage of mydriatics and those with mature cataracts were excluded from the study.

### Surgical technique

**Fig. 1:**
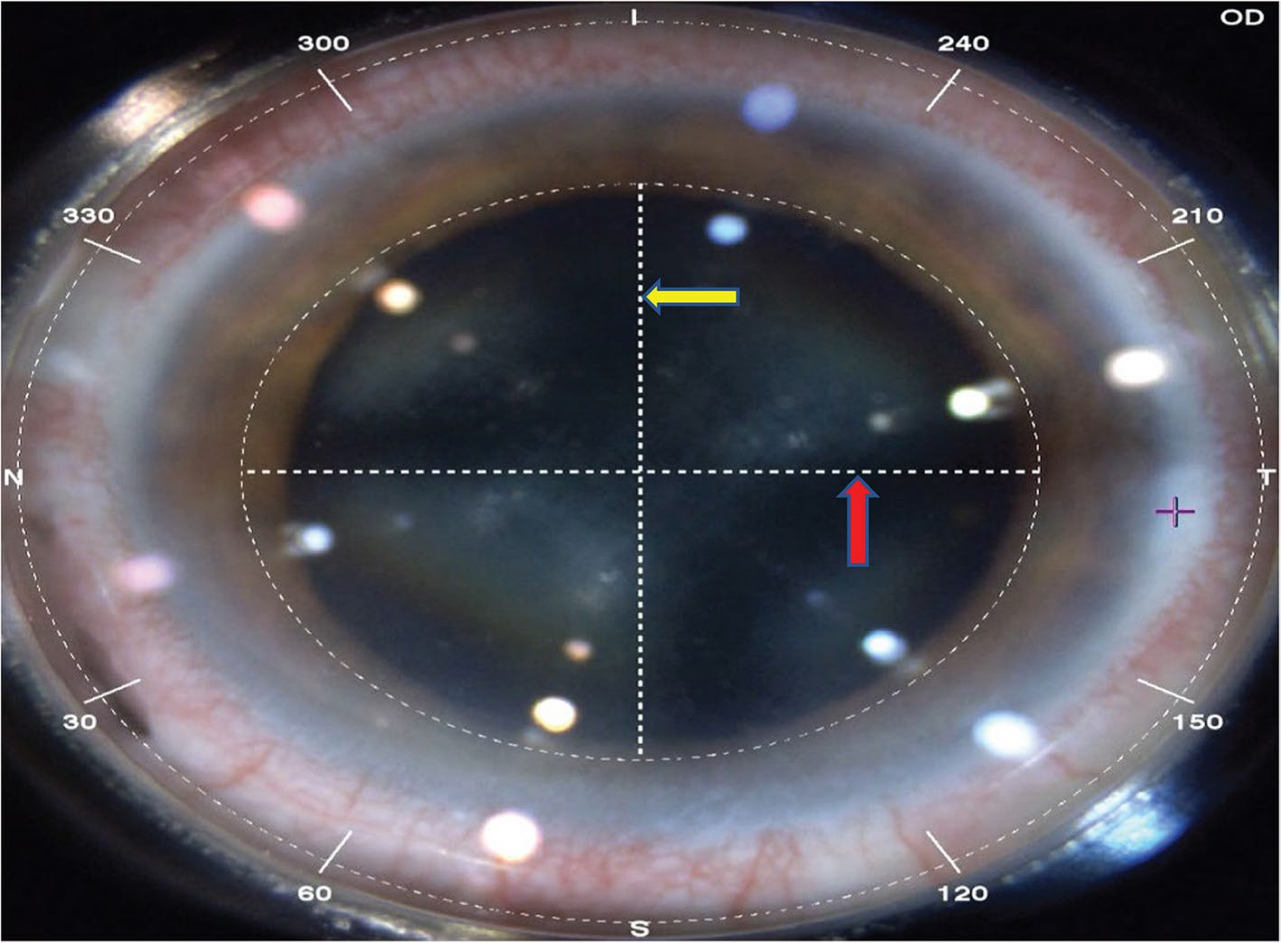
Image showing docking of the eye in LenSx. The surgeon ensured that the crossing point of the vertical line (90° to 270°) (yellow arrow) and the horizontal line (0° to 180°) (red arrow) matched with the center of the cornea

**Fig. 2:**
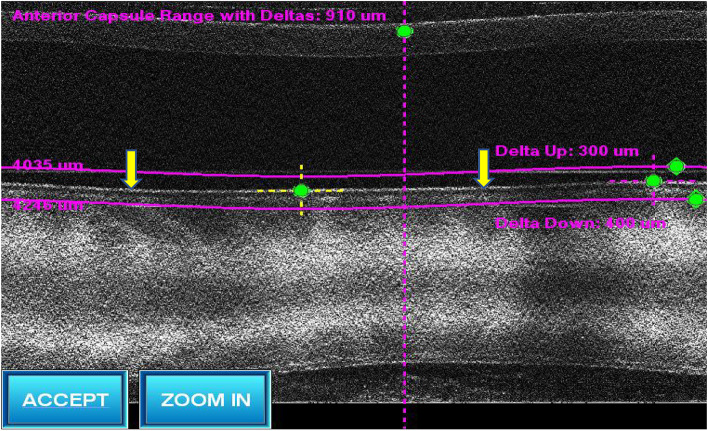
Cross-sectional image of the anterior capsule after docking in LenSx (yellow arrow). The surgeon ensured that cross sectional image of anterior capsule was as close to the horizontal as possible

**Fig. 3:**
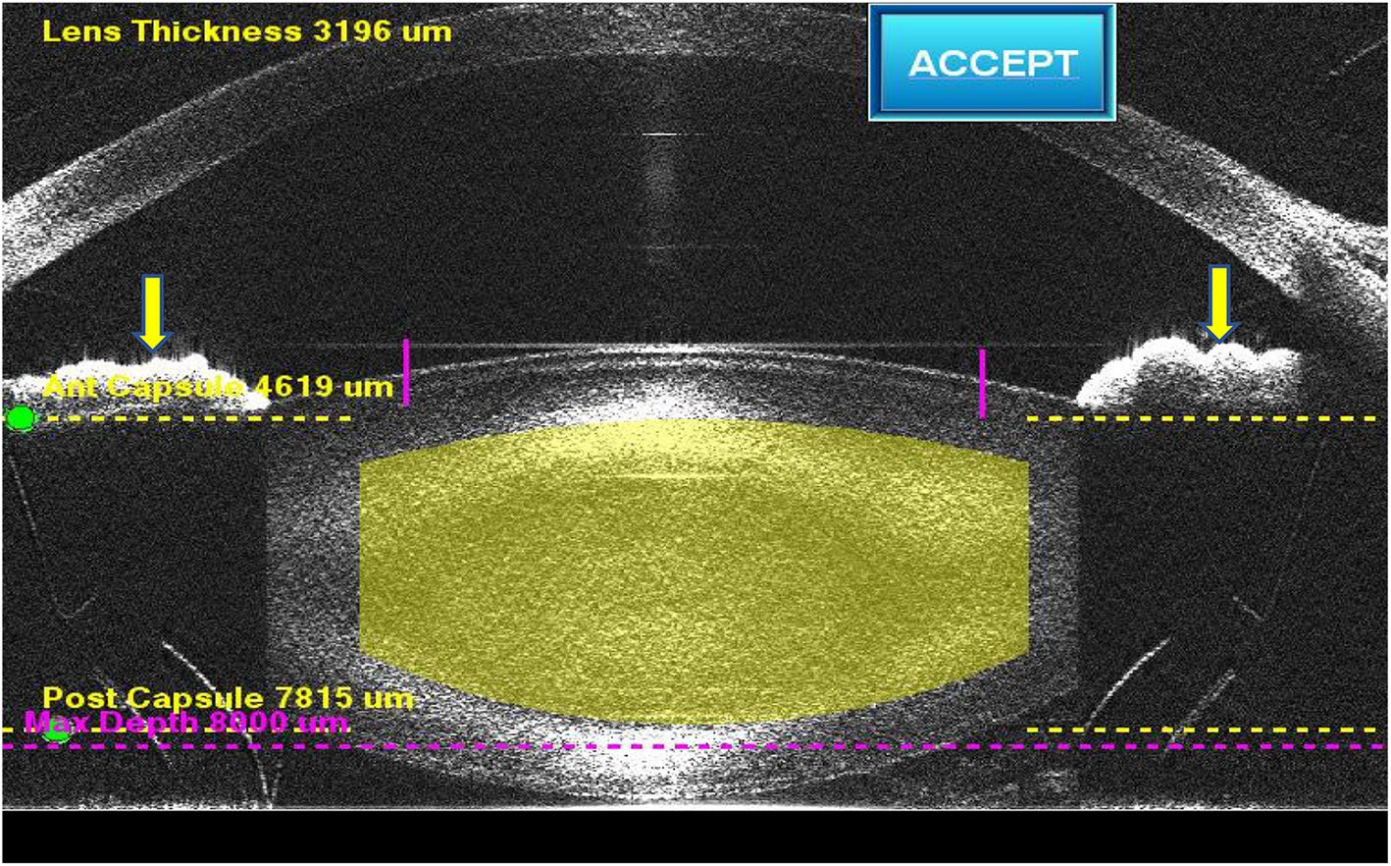
Cross-sectional image of iris after docking in LenSx (yellow arrow). The surgeon ensured that cross sectional image of iris was as close to the horizontal as possible

Moxifloxacin hydrochloride (0.5%) and 0 nevanac (0.1%) ophthalmic suspensions were administered four and two times a day, respectively, for three days prior to the operation. A single experienced surgeon performed all FLACS procedures with the LenSx platform (Alcon Laboratories, Inc., Fort Worth, TX, USA), to create a 5.3 mm capsulotomy centered on white-to-white using 8.0 mJ of energy (spot and layer separations: 9 μm each). Nuclear fragmentation was performed using the chop and cylinder technique with 8.0 mJ of energy (spot and layer separations: 9 μm each). The operating surgeon ensured docking quality and precision during FLACS by following these three steps: (i) the intersection of the vertical line (90° to 270°) and the horizontal line (0° to 180°) corresponded to the center of the cornea on the display (Fig. 1), (ii) the cross-sectional image of anterior capsule was as close to the horizontal as possible (Fig. 2), and (iii) the cross-sectional image of iris was as close to the horizontal as possible in the cross-sectional image of anterior segment optical coherence tomography in the LenSx platform (Fig. 3). Thereafter, a 2.2-mm corneal limbus incision and a 1.0-mm corneal side incision were made. The nucleus of the lens was then detached from the cortex by hydrodissection. Lastly, the AcrySof IQ IOL (model SN60WF; Alcon, Fort Worth, TX, USA) was placed accurately into the capsular bag. The ophthalmic viscoelastic device was completely aspirated, and a final check was made to ensure the closure of corneal incisions.

### Clinical data collection

All patients underwent a complete preoperative ophthalmic examination. Keratometry (K1, K2 and average K), anterior chamber depth (ACD), lens thickness (LT), anterior chamber width (ACW), white-to-white (WTW), curvature radius of anterior lens capsule (Front R) and axial length (AL) were measured using anterior segment swept-source optical coherence tomography device, CASIA 2 (Tomey Corp, Nagoya, Japan). The ACW was generated by CASIA 2 following automatic detection of the scleral spur and measurement of the distance between the scleral spur at 0º and 180º. Based on the acquired values of the above stated parameters, LT/ACW, LT/AL, LT/ACD and LT/ACW/Front R were calculated.

### Capsulorhexis size measurement

**Fig. 4:**
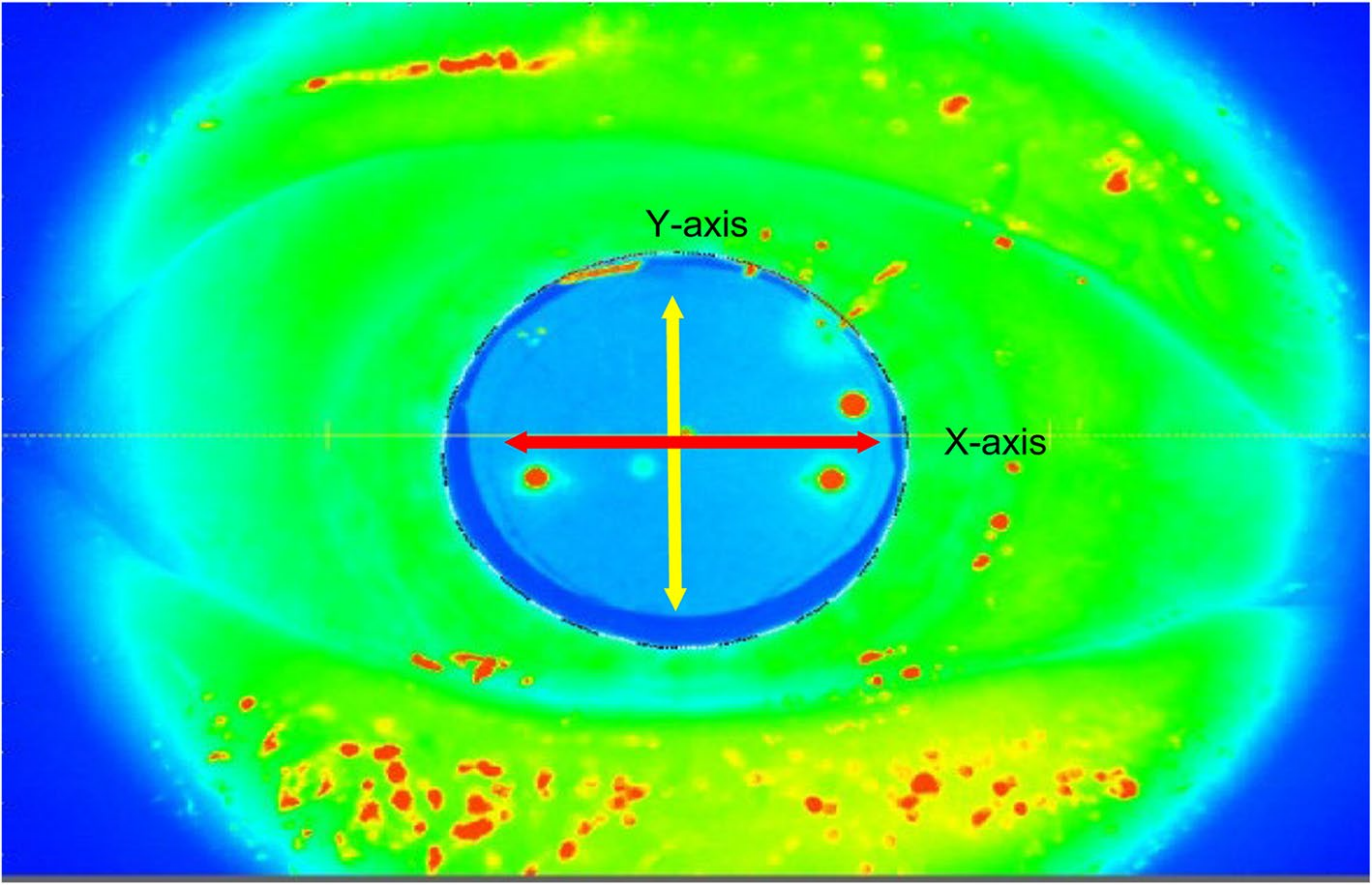
Frontal image of capsulorhexis after cataract operation. Capsulorhexis diameters (edge to edge) in X-axis (red arrow) and Y-axis (yellow arrow) were measured using the cross-sectional image

**Fig. 5:**
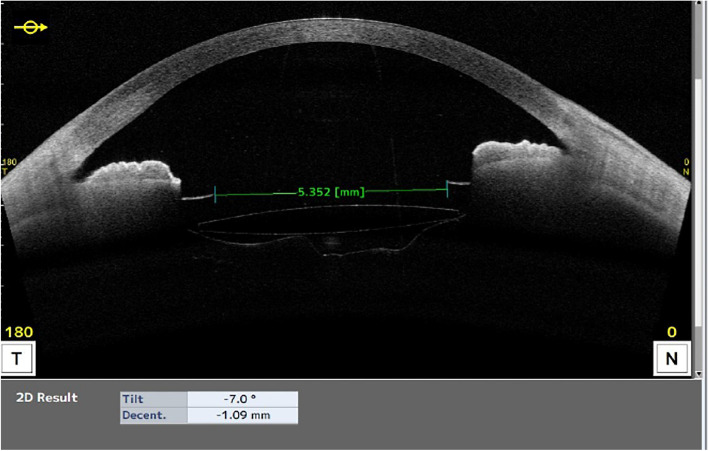
Cross-sectional image of capsulorhexis after operation in CASIA 2. Capsulorhexis diameters (edge to edge) in X-axis and Y-axis were measured using the cross-sectional image. Actual achieved capsulorhexis diameter (green line) was defined as the average of X-axis and Y-axis diameters

After completion of FLACS, patients were allowed to rest in a lounge for 10 to 15 min before being transferred to an examination room for ARCD mesurements using CASIA 2. All measurements were made by a single technician. Capsulorhexis diameters (edge to edge) in the X-axis and Y-axis were measured using the cross-sectional images generated by 3-dimensional optical coherence tomography in CASIA 2. Thereafter, the ARCD was calculated as the average of the X-axis and Y-axis diameters (Figs. 4 and 5). Further, the ratio of the programmed capsulorhexis diameter to the ARCD was defined as the capsulorhexis diameter index (CDI).

It is noteworthy that the LenSx platform utilizes the same technique of 3-dimensional optical coherence tomography for the measurement of programmed capsulorhexis size during FLACS. In addition to providing accurate, real-time measurements of capsulorhexis size, this technique guarantees that the measurements are free from the effects of corneal magnification and change in capsulorhexis position after cataract removal.

### Statistical analyses

To analyze the preoperative variables contributing to the CDI, univariate analysis was performed between the CDI and preoperative variables, including K1, K2, average K, ACD, LT, ACW, WTW, Front R, AL, LT/ACW, LT/AL, LT/ACD and LT/ACW/Front R. The Shapiro–wilk test (*p* < 0.05) was used to confirm that the CDI was non-normally distributed. Using Spearman’s rank correlation coefficient, we analyzed the correlations between the evaluated variables and CDI. The variables showing significant correlation were chosen as independent variables for the multiple linear regression analysis. Stepwise variable selection was performed to build the multivariable model. Analysis of variance was performed to assess the significance of the multivariate model and multicollinearity was checked using variance inflation factor (VIF). Additionally, Durbin-Watson test was done to detect the autocorrelation in the regression model's output. All statistical analyses were performed with SPSS software version 25.0 (IBM, Armonk, NY, USA), and *p* < 0.05 was considered as statistically significant.

Post hoc analysis　using G^*^power version 3.1.7 (University of Kiel, Germany) was conducted to determine whether the power of sample size was significant.

## Results

Sixty-seven eyes of 44 patients who received FLACS and IOL implantation were involved in this study. The measurements of evaluated ophthalmic characteristics of the patients are shown Table 1.　The programmed capsulorhexis dimeter was 5.3 mm for all eyes.　The average values of ARCD and CDI are shown Table 2.

**Table 1:** Results of ophthalmic measurements for evaluated eyes

	**Age** **(years)**	**ACD (mm)**	**K1** **(D)**	**K2** **(D)**	**Average K (D)**	**LT (mm)**		**ACW** **(mm)**	**WTW (mm)**	**Front R (mm)**	**AL** **(mm)**
Mean	70.5	2.45	44.57	43.92	44.25	4.71	11.44		11.57	9.37	23.43
SD	8.0	0.50	1.33	1.28	1.29	0.38	0.41		0.37	1.13	1.39
Maximum	89.0	3.63	49.60	47.58	48.59	5.39	12.13		12.38	11.86	27.53
Minimum	51.0	1.60	42.00	41.76	41.88	3.80	10.15		10.72	7.59	21.14

*ACD* Anterior chamber depth, *LT* Lens thickness, *ACW* Anterior chamber width, *WTW* White-to-white, *Front R* Curvature radius of anterior lens capsule, *AL* Axial length, *SD* Standard deviation

**Table 2:** Average values of ARCD and CDI for all operated eyes

	**ARCD (mm)**	**CDI**
Average value	5.30	1.00
SD	0.16	0.03
Maximum value	5.62	1.06
Minimum value	4.89	0.92

The programmed size of capsulorhexis was 5.30 mm in all cases

*ARCD* Actual resulting capsulorhexis diameter, *CDI* Capsulorhexis diameter index, *SD* Standard deviation

### Correlations between CDI and preoperative variables

ACD (*ρ* = 0.741, *p* < 0.001), LT (*ρ* = -0.906, *p* < 0.001), ACW (*ρ* = 0.369, *p* = 0.002), Front R (*ρ* = 0.855, *p* < 0.001), AL (*ρ* = 0.606, *p* < 0.001), LT/ACW (*ρ* = -0.911, *p* < 0.001), LT/AL (*ρ* = -0.909, *p* < 0.001), LT/ACD (*ρ* = -0.856, *p* < 0.001), LT/ACW/FrontR (*ρ* = -0.942, *p* < 0.001) showed significant correlations with CDI (Table 3). These nine variables were used as independent variables, while the CDI was used as the dependent variable in multiple linear regression analysis.

**Table 3:** Results of correlations between CDI and preoperative variables

	Correlations															
			CDI	ACD	K1	K2	Average K	LT	ACW	WTW	Front R	AL	LT/ACW	LT/AL	LT/ACD	LT/ACW/Front R
Spearmans's rho (ρ)	CDI	Correlation coefficient	1.000	.741^a^	0.079	0.107	0.090	-.906^a^	.369^a^	0.168	.855^a^	.606^a^	-.911^a^	-.909^a^	-.856^a^	-.942^a^
		Significance (2-tailed)		0.000	0.528	0.390	0.471	0.000	0.002	0.175	0.000	0.000	0.000	0.000	0.000	0.000

*CDI* Capsulorhexis diameter index, *ACD* Anterior chamber depth, *LT* Lens thickness, *ACW* Anterior chamber width, *WTW* White to white, *Front R* Curvature radius of anterior lens capsule, *AL* Axial length

^a^Correlation is significant at the 0.01 level (2-tailed)

### Multiple linear regression model

Front R and LT/ACW/Front R were selected as constants using stepwise variable selection. The multiple linear regression model was represented by the following equation:

CDI = 1.306–4.516 × LT/ACW/FrontR-0.011 × Front R, when *P* < 0.0001, adjusted R-squared = 0.919, VIF = 8.389, and Durbin-Watson ratio = 1.846.

Furthermore, the predicted postoperative capsulorhexis diameter (PPCD) equation was created on the basis of CDI equation as follows:

PPCD = programmed capsulorhexis diameter × 1.306–4.516 × LT/ACW/FrontR-0.011 × Front R.

### Power analysis

The results of the power analysis were as follows: effect size (f2) = 11.34, total sample size = 67, number of predictors = 9, probability of type 1 error (α) = 0.05, and power (1-β) = 1.00.

## Discussion

In recent decades, studies have been conducted to investigate the long-term effects of relationship between the lens capsule and IOL, factors contributing to the development of posterior capsular opacification (PCO) and anterior capsular contraction syndrome (ACCS), as well as the effects of IOL tilt and decentration on postoperative refraction [1, 2, 14–18]. Achieving patient satisfaction involves more than just creating the capsulorhexis without complications; it also requires consideration of changes in the postoperative refraction resulting from the procedure and reproducibility of the capsulorhexis size. Attaining a consistently sized capsulorhexis is vital due to several reasons. First, it has been reported that complete coverage of IOL edge with capsulorhexis is crucial to avoid the development of PCO, even more important than IOL edge design and material [14]. Hollick et al. reported PCO rates of above 50% in cases where IOL optics were inadequately covered with capsulorhexis [2]. They insisted that a capsulorhexis size of no more than 5.5 mm is desirable to prevent the development of PCO [2]. In addition, Gu et al. demonstrated incomplete capsulorhexis–IOL overlap as a risk factor for PCO [15]. Aykan et al. also reported a significant reduction in the incidence of PCO with complete capsulorhexis-IOL overlap [1]. A possible mechanism behind this observation may be that complete capsulorhexis-IOL overlap can contribute to bioadhesion of the IOLs and prevent proliferation and immigration of lens epithelial cells [1].

The second benefit of full overlap of capsulorhexis with the IOL optic edge is to lock the lens in the final effective lens position. Li et al. demonstrated that the size and complete coverage of IOL optics had statistically significant influence on effective position of the IOL as well as the postoperative refractive outcomes. They concluded that precision and reproducibility of capsulorhexis can minimize the postoperative refractive shift and aid in the effective positioning of intraocular lens, further enhancing patient satisfaction [5]. Large or asymmetric capsulorhexis can result in incomplete capsulorhexis-IOL overlap, allowing postoperative contractile forces of capsule to shift the IOL anteriorly to an unpredictable location or to cause IOL tilt and decentration [16–18]. Tilt and decentration of IOLs can cause deviation from expected postoperative refraction and undesirable visual aberrations [19, 20]. On the other hand, small capsulorhexis can also be problematic since it can increase the possibility of anterior capsule contraction inducing IOL tilt and decentration [5, 21]. It has been reported that even 0.3 mm dislocation of an aspheric IOL can lead to increased high order aberrations, which affect the quality of vision, particularly when using an aspheric multifocal or toric IOL [21, 22]. Additionally, with diffractive multifocal IOLs, if the capsulorhexis is too small, it can decrease the amount of light passing through the peripheral refractive zones. According to Kasper et al., the capsulorhexis size should be greater than 5.0 mm to maximize the benefits of aspheric IOLs [21]. Sugimoto et al. demonstrated that a small CCC size could induce rapid postoperative contraction of the anterior capsule [23]. It has been reported previously that a 5.0 mm capsulorhexis contracts to 4.4 mm on average within three months of the operation. On the other hand, an initial CCC size of more than 5.5 mm resulted in a final diameter of greater than 5.0 mm on an average [24, 25]. Thus, it is evident that multiple studies in the past have reported on the influence of precision and reproducibility of capsulorhexis on postoperative visual outcomes after cataract operation.

However, manual procedure has limited ability in terms of reproducibility and accuracy of capsulorhexis. Through their work, Kránitz et al. revealed that compared to the manual procedure, FLACS can create an accurately sized and precisely centered capsulorhexis, leading to better overlap of parameters, which was helpful in sustaining proper positioning of IOLs [6]. Nagy et al. also stated that FLACS created capsulorhexis can cover IOL optic edge more precisely and constantly, allowing better IOL centration than manual procedure, whereas partially covered IOL optic can induce myopization [7]. While more precise and reproducible capsulorhexis in FLACS is a well-accepted advantage over the manual procedure, eye surgeons have discovered differences between the ARCD and programmed capsulorhexis diameter by comparing the approximate capsulorhexis size with the size of an IOL’s optic. In fact, deviation of the ARCD from the programmed diameter in FLACS has been reported in some studies. Tackman et al. reported a mean deviation of 0.16 ± 0.17 mm from the programmed diameter [10]. Likewise, Nagy et al. demonstrated that the ARCD was 4.52 ± 0.2 mm when the programmed diameter was 4.50 mm [11]. The deviation of ARCD in the present study was similar to that observed in the aforementioned studies. ARCD was 5.30 ± 0.16 mm when the programmed diameter was 5.30 mm, and the range was from 5.62 mm to 4.89 mm. Although the degree of deviation in FLACS is relatively small, as previously stated, the smaller the deviation, the greater the benefit of premium IOLs. Packer et al. reported that a 5.25 mm capsulotomy optimized the prevention of PCO, consistency of the effective lens position and capsulotomy strength [26]. Therefore, it can be inferred that the range of ARCD in our study (5.62 mm to 4.89 mm) was clinically unsatisfactory. Presently, eye surgeons select the programmed capsulorhexis diameter based on their expertise. For instance, at our eye clinic, based on our prior experiences, we choose larger programmed diameter for eyes with thick lenses and shallow anterior chamber depth without certain scientific evidence to back this approach. As of yet, there exists no clinically reliable nomogram that can be followed to avoid oversized or undersized capsulorhexis. Therefore, the ARCD is not always optimal.

In the field of pediatric cataract surgery, there have been some studies that investigated the preoperative influential factors of ARCD. Dick et al. reported that age was the factor with maximum impact on the ARCD in pediatric cataract surgery, and that ARCD was larger in younger than older children [12]. They also created a formula to estimate ARCD in relation to the child’s age [12].

Furthermore, Liao et al. investigated the other factors that could affect ARCD in pediatric cataract surgery, including AL, ACD, K1 and K2. They found that AL and ACD were the most powerful influencing factors of ARCD by developing a multiple linear regression model [13]. However, as far as we know, no previous study has dealt with the development of multiple linear regression model for ARCD in the area of adult cataract surgery.

In this study, among the thirteen evaluated parameters, ACD, LT, ACW, Front R, AL, LT/ACW, LT/AL, LT/ACD, and LT/ACW/FrontR, showed significant correlation with CDI. Thereafter, LT/ACW/FrontR and Front R were selected via stepwise variable selection to build a multivariate model. Finally, a formula was created based on the multivariable model to calculate the PPCD.

Our results revealed that CDI showed significantly negative correlation with LT and positive correlation with ACW and Front R of the lens. Although the exact mechanism underlying these relationships requires further exploration, the formula presented by us might be able to assist eye surgeons in deciding a programmed capsulorhexis diameter that enables higher accuracy of prediction of CCC size.

There were some limitations to this study. First, although many preoperative variables were examined as possible influential factors, there may be other factors which can further improve the accuracy of the calculation formula. Second, measuring ACW and Front R requires anterior segment optical coherence tomography, which limits the widespread clinical use of this formula. A simpler and more accessible formula is needed to overcome this issue. Third, installation of the calculation formula into an intelligent software platform may be necessary so that programmed capsulorhexis diameter is automatically calculated just by inputting the LT, ACW and Front R. This will also help in avoiding human error and unnecessary miscalculation. Finally, further prospective studies are needed to confirm the accuracy of the formula developed in this study.

## Conclusions

Differences between the ARCD and programmed capsulorhexis diameter were frequently observed among adult patients who underwent FLACS in the present study. LT/ACW/Front R and Front R were found to be the most influential preoperative variables for ARCD and were used to develop a multiple linear regression model. The calculation formula created on the basis of the multivariable model may be useful in selecting optimal programmed capsulorhexis diameter to achieve more accurate ARCD.

## Data Availability

Most relevant datasets are included in this published article. Other datasets can be obtained from the corresponding author upon reasonable request**.**

## Abbreviations

ARCD: Actual resulting capsulorhexis diameter
ACCS: Anterior capsular contraction syndrome
ACD: Anterior chamber depth
ACW: Anterior chamber width
AL: Axial length
CDI: Capsulorhexis diameter index
CCC: Continuous curvilinear capsulorhexis
Front R: Curvature radius of anterior lens capsule
FLACS: Femtosecond laser-assisted cataract surgery
IOLs: Intra ocular lenses
LT: Lens thickness
PCO: Posterior capsular opacification
PPCD: Predicted postoperative capsulorhexis diameter
SD: Standard deviation
VIF: Variance inflation factor
WTW: White-to-white
